# Photobiont switching causes changes in the reproduction strategy and phenotypic dimorphism in the ***Arthoniomycetes***

**DOI:** 10.1038/s41598-018-23219-3

**Published:** 2018-03-21

**Authors:** Damien Ertz, Beata Guzow-Krzemińska, Göran Thor, Anna Łubek, Martin Kukwa

**Affiliations:** 1 Botanic Garden Meise, Department Research, Nieuwelaan 38, B-1860 Meise, Belgium; 2 Fédération Wallonie-Bruxelles, Direction Générale de l’Enseignement non obligatoire et de la Recherche scientifique, Rue A. Lavallée 1, B-1080 Bruxelles, Belgium; 3 Department of Plant Taxonomy and Nature Conservation, Faculty of Biology, University of Gdańsk, Wita Stwosza 59, PL-80–308 Gdańsk, Poland; 4 Department of Ecology, Swedish University of Agricultural Sciences, P.O. Box 7044, SE-750 07 Uppsala, Sweden; 5 Institute of Biology, The Jan Kochanowski University in Kielce, Świętokrzyska 15A, PL-25–406 Kielce, Poland

## Abstract

Phylogenetic analyses using mtSSU and nuITS sequences of *Buellia violaceofusca* (previously placed in *Lecanoromycetes*), a sterile, sorediate lichen having a trebouxioid photobiont, surprisingly prove that the species is conspecific with *Lecanographa amylacea* (*Arthoniomycetes*), a fertile, esorediate species with a trentepohlioid photobiont. These results suggest that *L*. *amylacea* and *B*. *violaceofusca* are photomorphs of the same mycobiont species, which, depending on the photobiont type, changes the morphology and the reproduction strategy. This is the first example of a lichenized fungus that can select between *Trebouxia* (*Trebouxiophyceae*) and trentepohlioid (*Ulvophyceae*) photobionts. *Trebouxia* photobionts from the sorediate morphotype belong to at least three different phylogenetic clades, and the results suggest that *Lecanographa amylacea* can capture the photobiont of other lichens such as *Chrysothrix candelaris* to form the sorediate morphotype. Phylogenetic analyses based on *rbcL* DNA data suggest that the trentepohlioid photobiont of *L*. *amylacea* is closely related to *Trentepohlia* isolated from fruticose lichens. The flexibility in the photobiont choice enables *L*. *amylacea* to use a larger range of tree hosts. This strategy helps the lichen to withstand changes of environmental conditions, to widen its distribution range and to increase its population size, which is particularly important for the survival of this rare species.

## Introduction

In lichen symbiosis, the mycobiont associates with a chlorophyll-containing partner, either an eukaryotic alga or a cyanobacterium, or both^1^. Recent studies revealed even more complex associations with the coexistence of bacteria, endolichenic fungi and basidiomycete yeasts with these primary partners^2–4^. Photobiont flexibility was recognized long ago by the existence of alternative cyanobacterial and green algal morphs (photomorphs or photosymbiodems) of the same mycobiont species^5,6^. In addition to differences observed in the colour of the thallus caused by the photobiont type, these photosymbiodems also differ in their thallus morphology, sometimes so significantly that, in the past, the chloromorph (with eukaryotic algae) and the cyanomorph (with cyanobacteria) were recognized as separate species, which were even sometimes classified in different genera^1,7^. Very rarely the photosymbiodems also differ in the additional development of asexual reproductive structures^1^. When a single fungal partner associates with a green alga and a cyanobacterium simultaneously, the latter photobiont type is located in distinct, usually easily morphologically recognizable areas of the thallus (i.e. cephalodia^8^), which in some species may enlarge and eventually form independent thalli^9^.

Switching between closely related eukaryotic green-algal strains is also frequently observed within a single lichen species^10–18^, but is not known to involve changes in the thallus anatomy and morphology. Recent molecular studies also highlight the coexistence of multiple green-algal taxa in a single lichen thallus^13,15,19,20^. A higher flexibility in photobiont choice is considered as a strategy to survive under new selective pressures and to widen the ecological niche amplitude enabling the occurrence even in nutrient-poor environments^1,12,21–24^, and may also drive the evolution of lichens^16,25^.

Some groups of crustose lichens reproduce mainly vegetatively by the formation of asexual propagules (usually soredia or isidia), whereas the development of ascomata (sexual reproduction) might be more or less limited to occasional events or are completely absent. In particular cases, the ability of the ascomata formation was completely lost in entire genera^26–28^. Determining the systematic position of sterile ascomycetes is difficult or impossible using only morphological or chemical characters of the thallus. In these cases the application of molecular markers and phylogenetic methods allows classification of those sterile samples within higher level taxonomic groups^26–28^.

Sterile lichens are often parts of so-called species pairs. This term, first introduced by du Rietz almost hundred years ago^29^, states that species pairs are two taxa that are morphologically and chemically very similar but differ in their predominant reproductive modes, i.e. “primary species” is fertile and reproduces sexually (by ascospores formed in apothecia), while “secondary species” is sterile and forms asexual diaspores (by isidia or soredia) (for review see^30^). Several hypotheses have been proposed to explain the presence of these species pairs and the most widely assumed one suggests that the majority of vegetatively reproducing taxa are members of the species pairs and evolved from the primary sexual taxon^29^. However, more recently Buschbom & Mueller^31^ proposed that the founding taxon of the species pair could be a species with either vegetative or sexual reproduction and switches between reproductive modes are triggered through symbiont interactions. Most phylogenetic studies of species pairs reported that neither sexual nor vegetative taxa are monophyletic, but rather intermingled and independent lineages did not correspond to reproductive mode^31–34^. However, none of these studies considered the impact of the photobiont type on the lichen reproduction strategy.

The lichen species *Buellia violaceofusca* G. Thor & Muhr is characterized by a sterile, thin, pale grey to almost white thallus producing dark brownish soralia with a violet tinge (but greenish when abraded), and the lack of detectable substances by thin-layer chromatography. Soralia are scattered, slightly elevated and sometimes confluent. This characteristic corticolous species contains a trebouxioid (green unicellular spherical cells) photobiont and was tentatively assigned to the genus *Buellia* De Not. due to its similarity with some species of that genus, especially the sorediate *B*. *griseovirens* (Turner & Borrer ex Sm.) Almb., and some other genera of *Lecanoromycetes*^35^. But because of the absence of apothecia, pycnidia and chemical compounds, the generic position was unclear. In order to verify the generic position of *B*. *violaceofusca*, fresh material was collected and sequenced. Here we show (1) that *B*. *violaceofusca* is conspecific with *Lecanographa amylacea* (Ehrh. ex Pers.) Egea & Torrente, (2) *L*. *amylacea* represents a first example of a lichen species that can switch between *Trebouxia* Puymaly and trentepohlioid photobionts, (3) a first example of dimorphism (thallus morphology and type of reproduction) resulting of the interchange between two different eukaryotic green algae taxa for a single mycobiont species, (4) *Trebouxia* photobionts from the sorediate morphotype known as *B*. *violaceofusca* belong to at least three different phylogenetic clades, (5) *Lecanographa amylacea* can capture the photobiont of other lichens such as *Chrysothrix candelaris* (L.) J.R.Laundon to form the sorediate morphotype, (6) the trentepohlioid alga of *L*. *amylacea* is most closely related to strains isolated from *Roccella* species, (7) photobiont switching in *L*. *amylacea* enables the lichen to widen its distribution range and to increase its population size.

## Results

### Fungal Phylogeny

**Figure 1:**
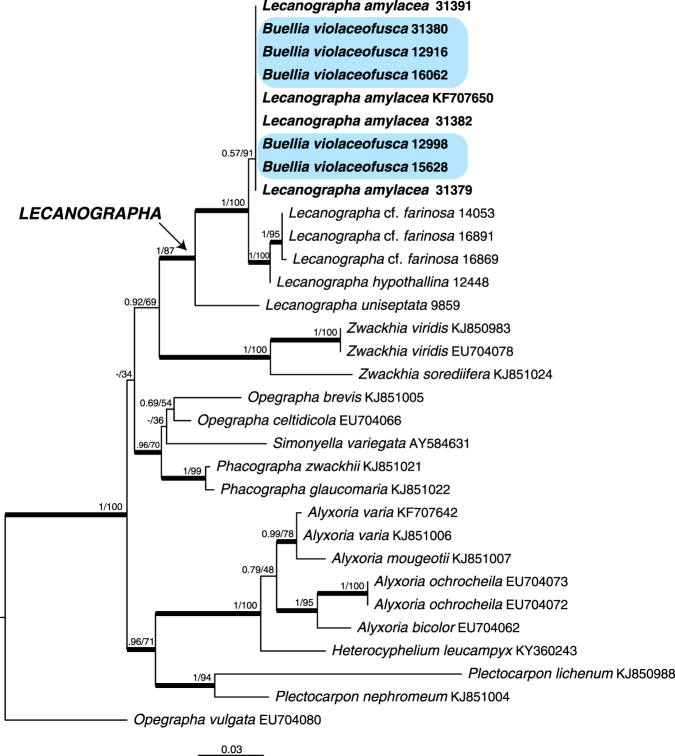
Phylogenetic relationships among samples within *Lecanographaceae* based on a data set of mtSSU sequences that resulted from a RAxML analysis. Posterior probabilities obtained from a Bayesian analysis and Maximum Likelihood bootstrap values are shown near the internal branches. Internal branches, considered strongly supported by both analyses, are represented by thicker lines. Collecting numbers of the authors following the species names of the newly generated sequences act as specimen and sequence identifiers, while those retrieved from GenBank are followed by their GenBank number. The samples of *Buellia violaceofusca* are highlighted.

**Figure 2:**
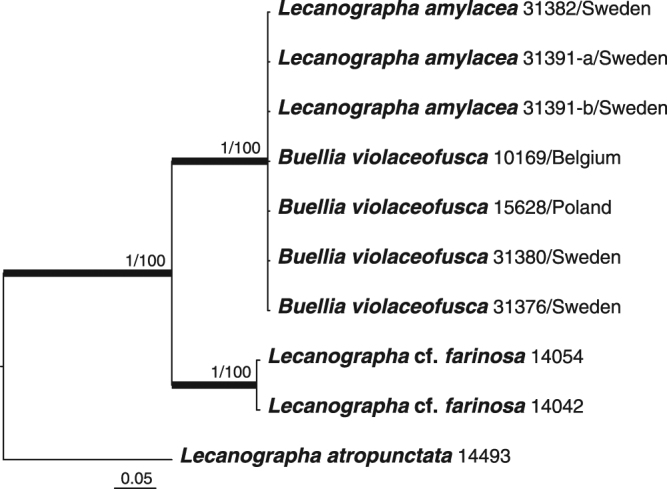
Phylogenetic relationships among samples within *Lecanographa* based on a data set of nuITS sequences that resulted from a Bayesian analysis. Posterior probabilities and Maximum Likelihood bootstrap values obtained from a RAxML analysis are shown near the internal branches. Internal branches, considered strongly supported by both analyses, are represented by thicker lines. All sequences were newly generated. Collecting numbers of the authors following the species names act as specimen and sequence identifiers.

The mtSSU dataset consisted of 32 specimens and 590 unambiguously aligned sites. In our mtSSU phylogenetic tree (Fig. 1), the *Lecanographaceae* are composed of four mainly supported lineages corresponding to the *Plectocarpon-Alyxoria* and *Phacographa-Opegrapha brevis* clades and the genera *Lecanographa* Egea & Torrente and *Zwackhia* Körb. The five specimens of *Buellia violaceofusca* are nested in the genus *Lecanographa* and intermixed with the specimens of *Lecanographa amylacea*. The nuITS dataset consisted of 10 specimens and 637 unambiguously aligned sites. In our nuITS phylogenetic tree (Fig. 2), the four specimens of *Buellia violaceofusca* cluster in a polytomy with the three specimens of *L*. *amylacea* because the nuITS sequences of these seven specimens are identical. These results prove that *B*. *violaceofusca* is conspecific with *L*. *amylacea*. Therefore, *B*. *violaceofusca* is synonymized with *L*. *amylacea*. *L*. *amylacea* is morphologically very different from *B*. *violaceofusca* notably by a thick, white-powdery thallus often dotted with brown flecks, containing a trentepohlioid photobiont (i.e. filamentous algae containing large amounts of carotenoid pigments, causing the algae to appear yellow orange in color), the absence of soralia and the occasional presence of apothecia. Our results prove thus that *B*. *violaceofusca* and *L*. *amylacea* represent two photomorphs of the same ascomycete species. These morphotypes are so distinct that they were described as two different species belonging to two different classes (*Lecanoromycetes* versus *Arthoniomycetes*).

### Algal phylogenies

**Figure 3:**
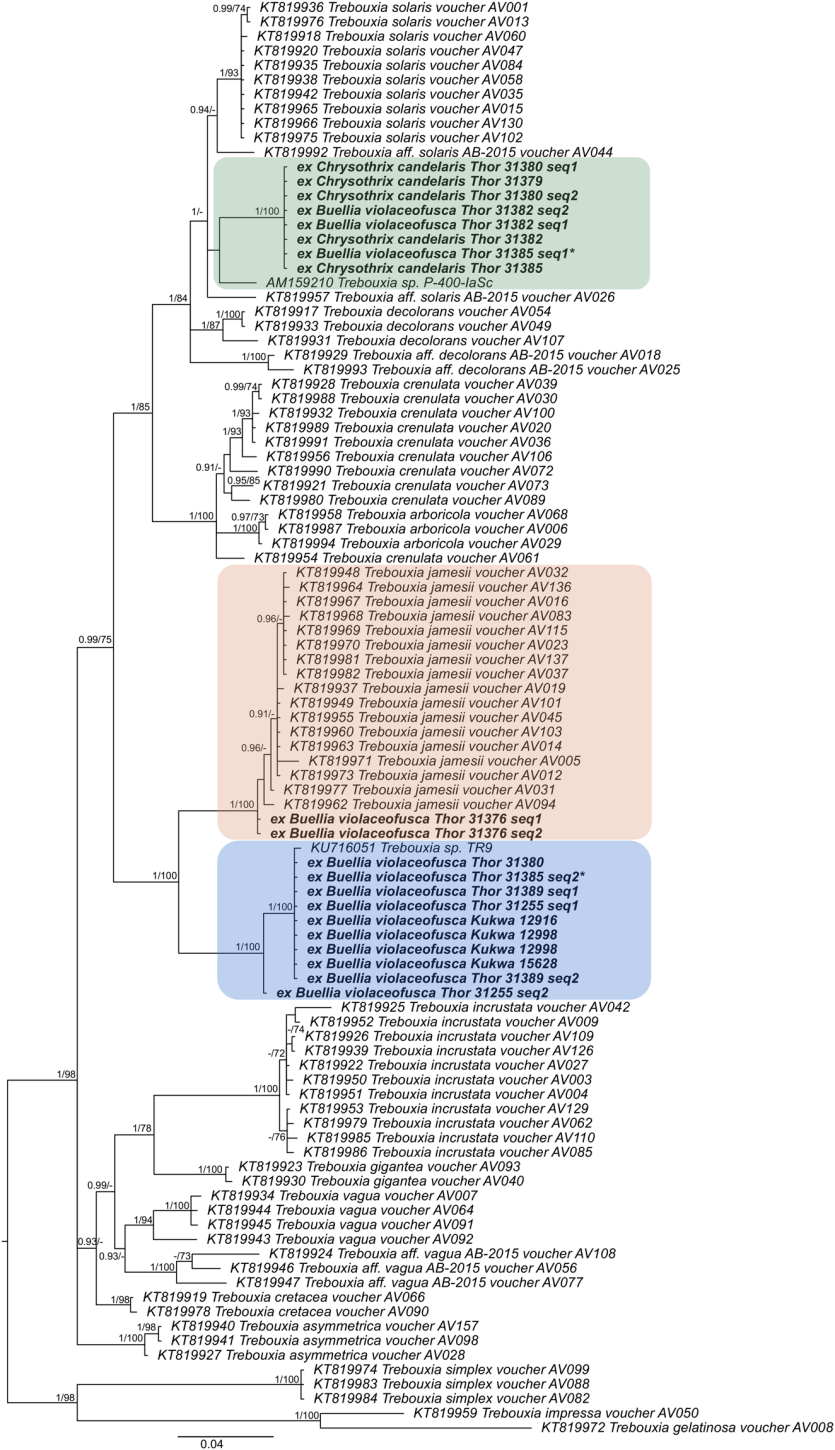
Phylogenetic relationships of *Trebouxia* photobionts from *Lecanographa amylacea* and *Chrysothrix candelaris* based on Bayesian analysis of nuITS dataset. Posterior probabilities and Maximum Likelihood bootstrap values obtained from a RAxML analysis are shown near the internal branches. Newly generated sequences are in bold, with collecting numbers of the authors following the species names. In case of specimens for which more than one sequence was generated the collecting numbers are followed with the number of sequence (i.e. seq. 1 or seq. 2). Specimen of ‘*Buellia violaceofusca’* morphotype Thor 31385 is marked with asterisk because two completely different *Trebouxia* strains were identified in its thallus. GenBank accession numbers of sequences retrieved from GenBank precede the species names. Clades with photobionts from *Lecanographa amylacea* and *Chrysothrix candelaris* are highlighted.

The *Trebouxia* algal dataset consisted of 99 nuITS sequences and 651 unambiguously aligned sites. The Bayesian tree (Harmonic mean was −4936.69) is shown in Fig. 3 with added bootstrap supports from RaxML analysis (ML Optimization Likelihood was −4490.471038). The newly sequenced photobionts revealed in three different clades within *Trebouxia* spp. The most common photobiont in *B*. *violaceofusca* thalli is *Trebouxia* sp. that groups together with *Trebouxia* sp. strain TR9 previously reported from *Ramalina farinacea* (L.) Ach.^19^. It was found in all Polish specimens (Kukwa 12916, 12998 and 15628) and four specimens from Sweden (i.e. Thor 31255, 31380, 31385 and 31389). A second lineage is related to the *T*. *solaris* clade and consists of *Trebouxia* sp. found in two Swedish specimens (i.e. Thor 31382 and 31385) as well as in four *Chrysothrix candelaris* specimens (i.e. Thor 31379, 31380, 31382, 31385) growing together with ‘*B*. *violaceofusca’*-morphotype. These molecular data strongly support our hypothesis that *Lecanographa amylacea* can capture the photobiont of *Chrysothrix candelaris* to form ‘*Buellia violaceofusca*’ thalli. This possibility was also concluded from the morphological observations of our specimens, because of the presence of *Buellia violaceofusca* at the margin of *Lecanographa amylacea* (with trentepohlioid photobiont) thalli in contact with *Chrysothrix candelaris*. A third lineage consists of two sequences from a specimen of *Buellia violaceofusca* (Thor 31376) that are nested within the *Trebouxia jamesii* clade. The latter is a common photobiont reported from e.g. *Candelariella vitellina* (Hoffm.) Müll. Arg., *Lecanora* spp., *Protoparmelia* sp., *Ramalina* spp., *Rhizocarpon geographicum* (L.) DC., *Rhizoplaca* sp.^36,37^. Interestingly, among the studied samples, we found that two different *Trebouxia* photobionts may be present in *Buellia violaceofusca* thalli. In specimen Thor 31385, we detected *Trebouxia* sp. TR9 and *Trebouxia* sp. related to *T*. *solaris* clade (Fig. 3).

**Figure 4:**
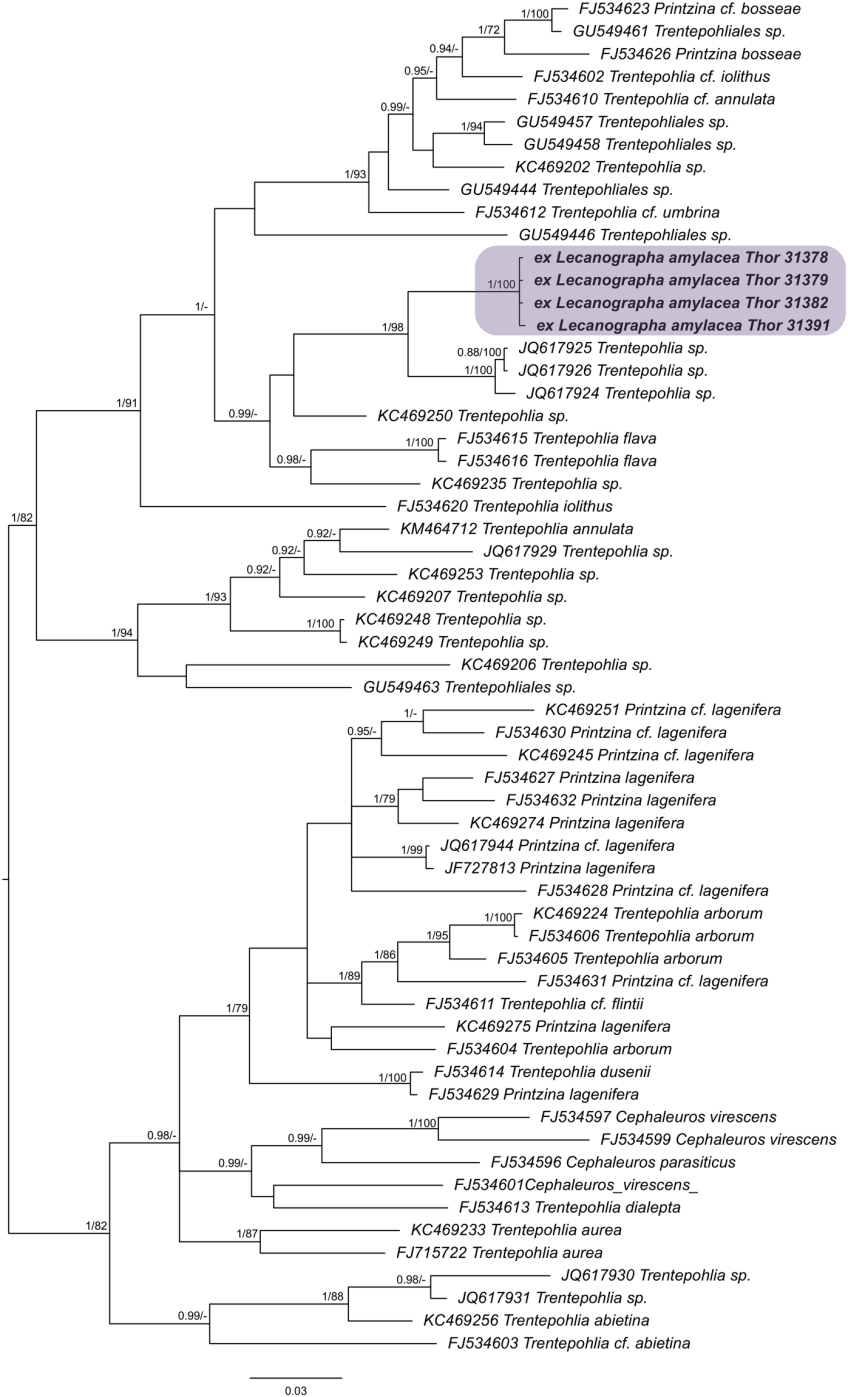
Phylogenetic placement of trentepohlioid photobionts from *Lecanographa amylacea* based on Bayesian analysis of chloroplast *rbcL* dataset. Posterior probabilities and Maximum Likelihood bootstrap values obtained from a RAxML analysis are shown near the internal branches. Newly generated sequences are in bold, with collecting numbers of the authors following the species names. GenBank accession numbers of sequences retrieved from GenBank precede the species names. Clade with photobionts from *Lecanographa amylacea* is highlighted.

The *rbcL* dataset of trentepohlioid photobionts consisted of 60 sequences and 668 unambiguously aligned sites. The Bayesian tree (Harmonic mean was −9624.90) is shown in Fig. 4 with added bootstrap supports from RaxML analysis (best tree ML = −8571.5689). The four sequenced specimens of *Lecanographa amylacea* (i.e. Thor 31378, 31379, 31382, 31391) form a lineage closely related to *Trentepohlia* photobionts isolated from *Roccella* spp. (JQ617924 from *R*. *linearis*; JQ617925 from *R*. *maderensis*; JQ617926 from *R*. *tinctoria*) collected in Spain and Portugal and reported by Hametner *et al*.^38^ as belonging to clade R. However, their proper identification to the species level is not possible due to insufficient data resolution in GenBank for this group of photobionts. The most closely related algae identified to the species level is a free-living *Trentepohlia flava* (Kützing) De Toni that was isolated from bark^39^.

### Taxonomy

**Figure 5:**
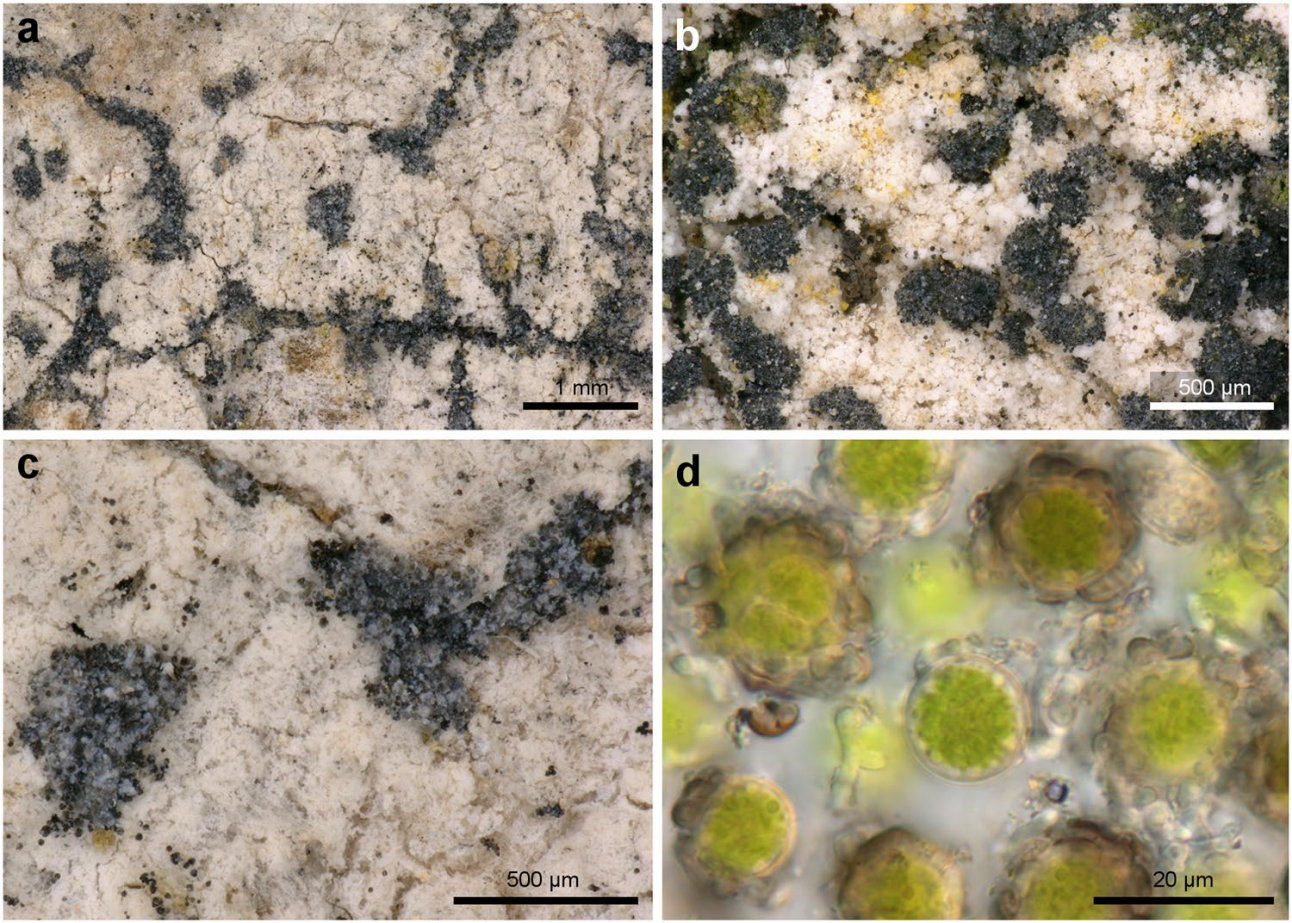
The sorediate morphotype of *Lecanographa amylacea* (previously recognized as *Buellia violaceofusca*). (**a**–**c**), soralia (**a**,**c** Ertz 10169; **b**: Ertz 16062); (**d**) trebouxioid algae (Thor 31382).

**Figure 6:**
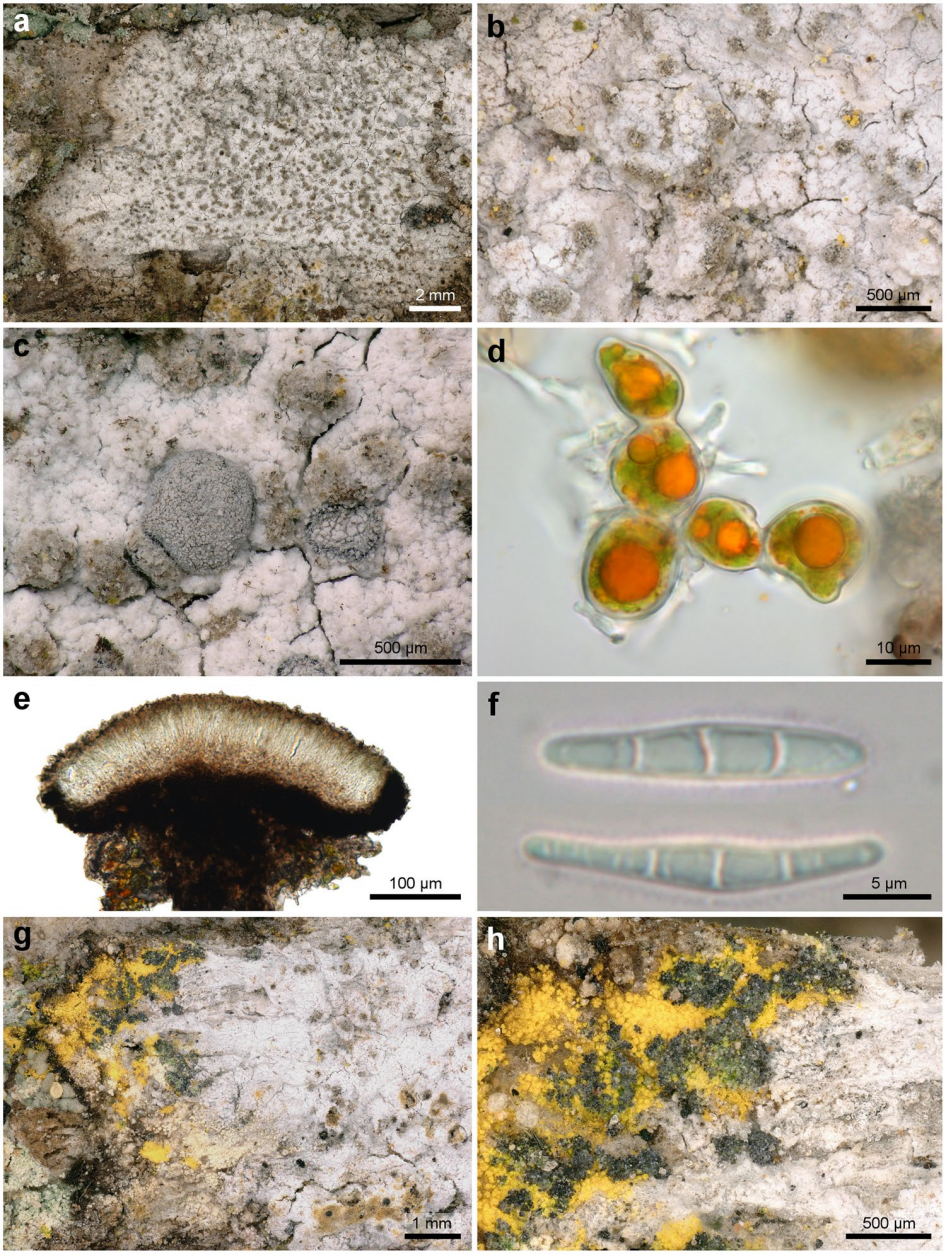
*Lecanographa amylacea*. (**a,b**) Thallus of the non-sorediate morphotype of *Lecanographa amylacea* (**a** Thor 31378; **b**, Thor 31379); (**c**) two apothecia (Thor 31378); (**d**) trentepohlioid algae (Thor 31382); (**e**) section of apothecium (Thor 31382); (**f**) ascospores in water (Thor 31378); (**g**,**h**), thallus of the non-sorediate morphotype of *Lecanographa amylacea* on the right (=white thallus), with the sorediate morphotype (=dark soralia with a greenish to violet tinge) present at the contact with *Chrysothrix candelaris* (=yellowish sorediate thallus) on the left (Thor 31391).

***Lecanographa amylacea*** **(Ehrh**. **ex Pers**.**) Egea & Torrente** (Figs 5, 6) *Bibliotheca Lichenologica* **54**: 122 (1994). Bas. *Lichen amylaceus* Ehrh. ex Pers., *Plant*. *Cryptog*. *Exsicc*. n°303 (1793)/*Ann*. *Bot*. *(Usteri)* **5**: 18 (1794). Type: (Germany), Hannoverae, Ehrh. sub *Lichen amylaceus* 303 (W, lectotype designated by Egea & Torrente^40^, not seen).

**Syn**. **nov**. *Buellia violaceofusca* G. Thor & Muhr, *Lichenologist* **23:** 11 (1991). Type: Sweden, Värmland, Visnums Kil par., Nötön Nature reserve, Arskagen, near Lake Vänern, 59°04′N, 14°01′E, *c*. 50 m, on the northern side of old *Quercus robur* in deciduous wood, 27 May 1985, L.-E. Muhr 7911 (UPS-holotype!, S-isotype!).

Sequenced specimens of esorediate morphotype of *Lecanographa amylacea*. **Sweden:** Västmanland, Kungsör parish, 3 km NNW Kungsör town, Jägaråsen, E of dirt road, 20 m, 59°27.036′N, 16°04.613′E, on dying, large *Quercus robur* in open, grazed deciduous forest, with *Buellia violaceofusca*, 25 July 2015, Thor 31379 (UPS); ibidem, 59°26.808′N, 16°04.754′E, on large *Quercus robur* in open, grazed deciduous forest, with *Buellia violaceofusca*, Thor 31382 (UPS); ibidem, Rytterne parish, 1.2 km SW Tidö Castle, 2 m S the dirt road, 59°30.073′N, 16°27.558′E, on large, exposed *Quercus robur*, Thor 31391 (UPS).

Sequenced specimens of sorediate morphotype of *Lecanographa amylacea* (originally identified as *Buellia violaceofusca*). **Belgium**: Ardennes, Wellin, à 4 km au sud de Chanly, ruisseau le Glan, 260 m, sur *Fraxinus* en forêt de feuillus, 29 September 2007, Ertz 10169 (BR); ibidem, St-Hubert, vallée de la Masblette, en amont du Pont Mauricy, 320 m, forêt de feuillus mixte de fond de vallée, sur un gros tronc de *Fraxinus*, 13 February 2011, Ertz 16062 (BR). **Poland:** Równina Bielska, Białowieża Primeval forest, Białowieża National Park, forest section no256, plot F07, *Querco-Piceetum*, on *Quercus robur*, May 2015, Kukwa 15628 & Łubek (BR, UGDA); plot A11, *Circaeo-Alnetum*, on bark of fallen *Fraxinus excelsior*, August 2014, Kukwa 12916 & Łubek (UGDA); plot M10, *Circaeo-Alnetum*, on bark of snag of dead *Fraxinus excelsior*, October 2014, Kukwa 12998 & Łubek (UGDA). **Sweden:** Uppland, S of Uppsala, Alsike parish, 300 m NW of Krusenberg mansion, 15 m, 59°44′13.62′′N, 17°38′37.51′′E, dense, grazed deciduous forest, on old large *Quercus robur*, 27 June 2015, Thor 31376 (BR, UPS); Västmanland, Kungsör parish, 3 km NNW Kungsör town, Jägaråsen, E of dirt road, 20 m, 59°26.879′N, 16°04.711′E, on large *Quercus robur* in open, grazed deciduous forest, 25 July 2015, Thor 31380 (UPS).

Additional sequenced specimens for the photobionts of *Lecanographa amylacea* (both morphotypes) and/or associated *Chrysothrix candelaris*. **Sweden:** Södermanland, Hölö parish, 1 km NW Tullgarn Castle, E of dirt road, 20 m, 58°57.467′N, 17°34.463′E, on one of two nearby (5 m) very large *Quercus robur* in open, grazed, deciduous forest, 25 July 2015, Thor 31378 (UPS). Västmanland, Kungsör parish, 3 km NNW Kungsör town, Jägaråsen, E of dirt road, 20 m, 59°26.808′N, 16°04.754′E, on large *Quercus robur* in open, grazed deciduous forest, 25 July 2015, Thor 31382 (UPS); ibidem, 59°26.810′N, 16°04.779′E, Thor 31385 (UPS); Rytterne parish, 620 m SW Tidö Castle, 3 m N the dirt road, 25 m, 59°30.119′N, 16°28.176′E, on large, dead *Quercus robur* with almost no bark, 25 July 2015, Thor 31389 (UPS); Gotland, Fide parish, 1.2 km NNW Fide church, W of the road, the wooded meadow Fide annex, 10 m, 57°04.993′N, 18°18.588′E, on *Quercus robur* 287 cm in circumference, 21 May 2015, Thor 31255 & Knutsson (UPS).

## Discussion

Here we report *Lecanographa amylacea* as a first example of a lichen that uses either *Trebouxia* (Trebouxiophyceae) or trentepohlioid algae (Ulvophyceae) as primary photobiont. This is also the first example of a lichen with two very distinct photomorphs, both containing distantly related green algae. Indeed, none of the studies that previously recorded green algae switch within single lichen species suggested an impact on the thallus morphology (see introduction). The high degree of dimorphism involving alterations in the thallus anatomy and morphology was so far only known from lichens in which the same mycobiont associates with either a green alga or a cyanobacterium (e.g. *Dendriscocaulon*–*Lobaria*^5,7,8^). Moreover, photobiont switching between different species of green algae so far mainly concerned lichens belonging to the class *Lecanoromycetes*. One exception is *Coniocarpon cinnabarinum* DC., which belongs to the *Arthoniomycetes* and associates with the trentepohlialean algae *Printzina lagenifera* (Hildebrand) R.H. Thompson & Wujek and one of its close relative, probably belonging to the same genus^18^. However, no impact on the thallus appearance has ever been recorded before when the closely related green algae exchange in the same lichen species.

The two types of photobionts (trentepohlioid versus *Trebouxia*) with which *Lecanographa amylacea* associates are obviously responsible for the strong dimorphism observed. Once *L*. *amylacea* has gained one of the *Trebouxia* photobionts, the lichen species can disseminate by means of soredia that propagate jointly both symbionts and may form independent sorediate thalli previously recognized as *Buellia violaceofusca*. The *Trebouxia* photobiont species might be maintained in this sterile sorediate photomorph as this reproduction strategy should allow preservation of the relationship among symbionts. However, maintenance of the symbiotic associations seems to be rather an option than a strict consequence of joint symbiont dispersal in lichens^41^ and horizontal transmissions with other lichen species are possible as proved e.g. for the genus *Lepraria* Ach., which disperses solely by soredia-like granules^14^.

In addition to the alteration of morphology, the type of photobiont might influence the sexuality of the mycobiont. The *Trebouxia*-morphotype of *L*. *amylacea* is not known to form ascomata, contrary to its trentepohlioid morphotype, which is on the other hand never sorediate. When *L*. *amylacea* is fertile, ascospores, which disseminate only the fungal partner, could switch photobionts by the capture of *Trebouxia* algae from other lichens or from free-living, non-lichenized *Trebouxia* (despite these latter appear to be less frequently encountered), leading to the subsequent development into the sorediate morphotype. Buschbom & Mueller^31^ hypothesized that switches between reproductive modes are triggered through symbiont interactions. They suggested that the asexual reproductive mode is advantageous when the relationship between the mycobiont and the photobiont is optimal in a given environment allowing the rapid dispersion of both partners: in this situation, it is advantageous for the mycobiont not to switch algae. In disadvantageous environmental situations, sexual reproduction of the mycobiont is preferred because this would increase the chance to “escape” from its current partner. The sexual reproduction allows the mycobiont to acquire new partners as well as generates variability through recombination, with the development of new genotypes that may be better adapted to local and changing conditions. Similarly, Ellis & Coppins^42^ suggested that the asexually reproducing crustose lichens are better adapted to stable habitats. However, it is unclear if this might also be the case for the asexual/sexual morphotypes of *L*. *amylacea*. Studies at local and global scales should be performed to better understand which might be the combination of environmental factors that drive the asexual/sexual reproduction in *L*. *amylacea* and those responsible of the photobiont switches.

A higher flexibility in the photobiont choice is considered as a strategy to increase ecological tolerance, enabling the lichen to withstand changes of environmental conditions (e.g. shifts in light or humidity regimes), to colonize diverse microhabitats, and to widen its distribution range^8,12,16,21,22^. As a consequence, it increases the population size of the fungal partner^15^, which is particularly important for the survival of a rare species such as *L*. *amylacea*. Since its description from Sweden in 1991, ‘*Buellia violaceofusca*’ (=the *Trebouxia*-morphotype of *L*. *amylacea*) has been recorded from several European countries^43,44^. This morphotype is even present in regions where the trentepohlioid morphotype has never been recorded (e.g. Belgium or Białowieża Forest in Poland) suggesting that the *Trebouxia* photobionts may offer higher fitness in some habitats or in climatically different regions. The *Trebouxia* morphotype might also be more easily dispersed with successful new colonization as soredia are produced in large quantities and disseminate the fungal and algal symbionts together. The trentepohlioid morphotype of *L*. *amylacea* appears to be exclusively confined to old *Quercus* trees^40,45–48^, while the *Trebouxia*-morphotype can sometimes colonize other phorophytes, mainly *Fraxinus*^43,47–49^ and *Acer*^44,50^ in addition to *Quercus*. The use of a larger range of tree hosts is an obvious advantage that might explain the success of the *Trebouxia*-morphotype in some regions. In addition, the *Trebouxia*-morphotype has the capacity to colonize much younger *Quercus* trees than the trentepohlioid morphotype, the latter only occurring on *Quercus* over 200 years old with > 60 mm bark crevices as shown in Sweden^45,51,52^. As a consequence, the *Trebouxia*-morphotype has a wider ecological niche on *Quercus* trees compared to the trentepohlioid morphotype, with a much higher number of *Quercus* trees being thus suitable for colonization by the *Trebouxia*-morphotype^51,52^. Despite the ability to switch photobionts, *L*. *amylacea* (including ‘*B*. *violaceofusca*’) is rare throughout its distributional range, being often known from small populations (on a single or a few trees). With other epiphytic species occurring on old *Quercus* trees in semi-open landscapes, it disappears notably because of shading by developing secondary woodland^52^, but the *Trebouxia*-morphotype of *L*. *amylacea* appears to have the capacity to survive in more shaded conditions, and might thus replace the trentepohlioid morphotype in more dense forests. Studies on the forest types and of the environmental parameters, particularly light and humidity, which favour each morphotypes should be performed to better understand their distribution.

The identification of trentepohlioid algae in lichens are difficult on the basis of morphological features even at the generic level, especially as sexual stages of algae are usually suppressed in lichens^18^. As a consequence, we used molecular methods to investigate the identity of the trentepohlioid alga of *L*. *amylacea* (Fig. 4). Unfortunately, the taxonomic coverage in GenBank for this group of algae is still very limited and uneven, and many deposited sequences are from samples identified to the genus level or above, with many genera being polyphyletic. Brooks *et al*.^53^ summarized the most recent molecular studies and showed five well-supported lineages within *Trentepohliales*, of which none correspond to the morphologically defined genera. Lichenized trentepohlioid photobionts were found within most of these clades. It is estimated that approximately 23% of lichen-forming fungi associate with trentepohlioid algae^54^. In our study, the photobiont of *Lecanographa amylacea* was found to be closely related to *Trentepohlia* spp. isolated from different *Roccella* species (Fig. 4), which were shown to belong to clade R closely related to strains representing e.g. *Printzina bosseae* (De Wildeman) R.H.Thompson & Wujek, *Trentepohlia annulata* F. Brand, *T*. *iolithus* (L.) Wallroth^18^, and *T*. *flava* (Kützing) De Toni (Fig. 4). Because molecular studies only started to elucidate the identity and diversity of trentepohlialean photobionts involved in lichen symbiosis^18,38,54^, more work is needed to investigate the selectivity and specificity (as defined by Yahr *et al*.^12^, selectivity denotes the frequency of association among partners, whereas specificity denotes the phylogenetic range of associated partners) with this type of photosynthetic partner. Although previous studies suggested that at least some bark-inhabiting lichens may switch their trentepohlialean photobionts^18,54^, none of these studies showed photobiont switch from coccoid green algae to trentepohlioid photobiont.

The most common trebouxioid photobiont detected in sorediate morphotype of *L*. *amylacea* thalli is *Trebouxia* sp. TR9 previously reported only from *Ramalina farinacea*^19^. In one of these thalli, the same photobiont as in *Chrysothrix candelaris* was detected together with *Trebouxia* sp. TR9 (Thor 31385; Fig. 3). Such multiple algal genotypes have been previously found in a single thallus of different species^13,15,20^, which may be beneficial in terms of lichen’s ability to respond to environmental changes or to occupy diverse microenvironments^22^. Moreover, we also found the *Trebouxia*-morphotype of *L*. *amylacea* to contain the same photobiont as found in the neighboring thallus of *C*. *candelaris* (Thor 31382; Fig. 3). This suggests that *Lecanographa amylacea* may obtain photobionts from different lichens, one of those being probably *Chrysothrix candelaris*. In the specimen Thor 31382, ‘*B*. *violaceofusca*’-like soralia were present along the margins of the trentepohlioid morphotype of *L*. *amylacea* thalli contiguous to those of *Chrysothrix candelaris*, without borders between them (see also Fig. 6g,h for specimen Thor 31391). It suggests that the growing thallus of *L*. *amylacea* is able to capture the photobiont from *C*. *candelaris* by entering its neighboring thalli (so called ‘horizontal transmission’ allowing acquisition of new symbionts). Moreover, thalli of *C*. *candelaris* often become paler and appear to be killed in close vicinity of the ‘*B*. *violaceofusca*’-morphotype thalli. Phenomenon in which the mycobiont captures the photobiont of other lichen species has been already described long ago (^10^for *Diploschistes muscorum* (Scop.) R. Sant. in the *Ostropales* in *Lecanoromycetes*). However, it is known that photobionts with identical nuITS sequences are present in lichens growing within the same lichen community, without necessarily implying that the photobiont was taken over from other lichens, as free-living *Trebouxia* can also be captured^55^. A study of the photobionts of entire lichen communities as well as their free-living algae will be needed to identify the different photobiont sources that might explain the existence of the multiple trebouxioid-algal taxa detected in the ‘*B*. *violaceofusca*’-morphotype.

Composite or joint thalli of the two morphotypes of *L*. *amylacea* have not been reported so far thus explaining why connection between the two previously recognized taxa, *B*. *violaceofusca* and *L*. *amylacea*, has never been suggested before, nor a possible placement of the former one in the *Arthoniomycetes*. Both morphotypes have even been used as different indicator species of old trees in several ecological studies^51,52,56^.

*Lecanographa amylacea* belongs to the *Lecanographaceae*, a family of species that are either lichenized with a trentepohlioid photobiont or that are lichenicolous^57,58^. The presence of a morph with *Trebouxia* algae in this family is therefore surprising. Interestingly, *Lecanographa insolita* Lendemer & K. Knudsen was also recently described as having a trebouxioid photobiont^59^. However, this lichen has never been found fertile and has not been sequenced, so the species was only tentatively assigned to the *Lecanographaceae* on basis of secondary chemistry and of the anatomy of immature ascomata observed in only one specimen. Its taxonomic affiliation should be verified by sequencing to determine whether it is a second example of trebouxioid photomorph in the family. Species of *Arthoniales* having unicellular green algae are otherwise only known in the *Arthoniaceae* (e.g. *Arthonia phlyctiformis* Nyl.) and the *Chrysotricaceae* (e.g. *Chrysothrix candelaris*)^58^. Molecular studies are needed to determine the identity of the green unicellular algae living in symbiosis with *Arthoniales*, our study being the first step in this field. *L*. *amylacea* represents an interesting model to explore the evolutionary mechanisms that generate fungal specificity and selectivity in the *Arthoniomycetes* and lichens in general.

## Methods

### Amplification and sequencing

Well-preserved and freshly collected specimens lacking any visible symptoms of fungal infection were used for DNA isolation. Genomic DNA was isolated from fresh specimens using the CTAB extraction protocol (for the specimens of *Lecanographa atropunctata* Sparrius, Saipunk. & Wolseley, *L*. cf. *farinosa* (Hepp) Egea & Torrente, *L*. *hypothallina* (Zahlbr.) Egea & Torrente and *L*. *uniseptata* Ertz, van den Boom, Tehler & Degreef) or modified CTAB protocol^60^ (for the specimens of *Buellia violaceofusca* Kukwa 12916, 12998). Hand-made sections of the hymenium (e.g. *Lecanographa amylacea*, Thor 31382), or the thallus (e.g. *L*. *amylacea*, Thor 31379, 31391), as well as soredia (specimens of *Buellia violaceofusca* and *Chrysothrix candelaris*) were also used for direct PCR as described in Ertz *et al*.^61^. For amplification of *Trebouxia* photobionts one or two reactions from the same thallus were performed.

Amplification reactions were prepared for a 50 µl final volume containing 5 µl 10 × DreamTaq Buffer (Thermo Scientific), 1.25 µl of each of the 20 µM primers, 5 µl of 2.5 mg mL^−1^ bovin serum albumin (Thermo Scientific), 4 µl of 2.5 mM each dNTPs (Thermo Scientific), 1.25 U DreamTaq DNA polymerase (Thermo Scientific), and 1 µl of template genomic DNA or, in case of direct PCRs, lichen fragments. For amplification of algal DNA (*Trebouxia* and *trentepohlioid* photobionts) StartWarm HS-PCR Mix (A&A Biotechnology) was used. A targeted fragment of about 0.8 kb of the mtSSU rDNA was amplified using primers mrSSU1 and mrSSU3R^62^ and a fragment of about 0.6 kb of the nuITS rDNA (ITS1 + 5.8 S + ITS4) using primers ITS1F and ITS4^63^. We also amplified a fragment of algal nuITS rDNA (ITS1 + 5.8 S + ITS4) using *Trebouxia*-specific primers AL1500bf ^64^ and ITS4M^13^ and a fragment of trentepohlioid chloroplast *rbcL* gene using the following primers a-ch-rbcL-203–5′MPN and a-ch-rbcL-991–3′-MPN^54^. The yield of the PCRs was verified by running the products on a 1% agarose gel using ethidium bromide. PCR products were purified using Clean-Up Concentrator (A&A Biotechnology). Both strands were sequenced by Macrogen® using amplification primers. Sequence fragments were assembled with Sequencher version 5.3 (Gene Codes Corporation, Ann Arbor, Michigan). Sequences were subjected to ‘megablast’ searches to verify their closest relatives and to detect potential contaminations (no contaminations were found).

### Phylogenetic analyses of fungi

Because the first mtSSU sequences obtained from *Buellia violaceofusca* from Belgium and Poland were identical based on BLASTN search to the single mtSSU sequence of *Lecanographa amylacea* present in GenBank (viz. KF707650), more samples of the two lichens were collected in Sweden and sequenced. In addition, mtSSU and nuITS sequences were also generated for four more species of *Lecanographa*. A total of thirteen new mtSSU sequences for this study were obtained for placing the newly sequenced samples in a phylogeny of the family (Table 1). Members representing all genera currently accepted in the *Lecanographaceae* and for which a mtSSU sequence was available, were selected from Ertz & Tehler^57^, Frisch *et al*.^58^ and Van den Broeck *et al*.^65^. In order to test if *B*. *violaceofusca* is conspecific with *L*. *amylacea*, we also sequenced a more variable locus i.e. 10 nuITS (ITS1 + 5.8 S + ITS4; Table 1). *Opegrapha vulgata* (Ach.) Ach. was chosen as the outgroup species for the mtSSU tree and *Lecanographa atropunctata* for the nuITS tree. The sequences were aligned using *MAFFT v6*.*814b*^66^ within Geneious and improved manually using Mesquite 3.04. No regions were excluded from the nuITS dataset. Ambiguous regions corresponding to a total of 371 aligned sites were delimited manually following Lutzoni *et al*.^67^ and excluded from the mtSSU dataset. These regions were mainly due to a long insertion in the sequence of *O*. *celtidicola* (positions 197 to 475 of the sequence EU704066), and to the regions corresponding to positions 410 to 441 and 677 to 687 in the sequence KJ851024 of *Zwackhia sorediifera* and positions 597 to 644 in the sequence EU704080 of *O*. *vulgata*.

**Table 1:** Species names, voucher specimens and GenBank accession numbers for the specimens included in the phylogenetic analyses and for which sequences were generated in this study. Sequences generated by direct PCR are indicated by an asterisk following the GenBank accession number.

Species name	Voucher	Accession numbers			
		Fungal mtSSU	Fungal nuITS	Algal nuITS	Algal *rbcL*
*Chrysothrix candelaris*	Thor 31379	—	—	MG687500*	—
*C. candelaris*	Thor 31380	—	—	MG687519* MG687501*	—
*C. candelaris*	Thor 31382	—	—	MG687504*	—
*C. candelaris*	Thor 31385	—	—	MG687506*	—
*Buellia violaceofusca*	Ertz 10169	—	MG838723*	—	—
*B*. *violaceofusca*	Ertz 16062	MG845003*	—	—	—
*B*. *violaceofusca*	Kukwa 12916	MG845004	—	MG687514	—
*B*. *violaceofusca*	Kukwa 12998	MG845005	—	MG687515 MG687516	—
*B*. *violaceofusca*	Kukwa 15628	MG845006*	MG838724*	MG687518*	—
*B*. *violaceofusca*	Thor 31255	—	—	MG687513* MG687512*	—
*B*. *violaceofusca*	Thor 31376	—	MG838725*	MG687510* MG687511*	—
*B*. *violaceofusca*	Thor 31380	MG845007*	MG838726*	MG687507*	—
*B*. *violaceofusca*	Thor 31382	—	—	MG687503* MG687502*	—
*B*. *violaceofusca*	Thor 31385	—	—	MG687505* MG687508*	—
*B*. *violaceofusca*	Thor 31389	—	—	MG687509* MG687517*	—
*Lecanographa* *amylacea*	Thor 31378	—	—	—	MG676235*
*L*. *amylacea*	Thor 31379	MG845008*	—	—	MG676236*
*L*. *amylacea*	Thor 31382	MG845009*	MG838727*	—	MG676237*
*L*. *amylacea*	Thor 31391-a	MG845010*	MG838728*	—	MG676238*
*L*. *amylacea*	Thor 31391-b	—	MG838729	—	—
*L*. *atropunctata*	Ertz 14493	—	MG838730	—	—
*L*. cf. *farinosa*	Ertz 14042	—	MG838731	—	—
*L*. cf. *farinosa*	Ertz 14053	MG845011	—	—	—
*L*. cf. *farinosa*	Ertz 14054	—	MG838732	—	—
*L*. cf. *farinosa*	Ertz 16869	MG845012	—	—	—
*L*. cf. *farinosa*	Ertz 16891	MG845013	—	—	—
*L*. *hypothallina*	Ertz 12448	MG845014	—	—	—
*L*. *uniseptata*	Ertz 9859	MG845015	—	—	—

Bayesian analyses were carried out on the fungal mtSSU and the nuITS datasets separately using the Metropolis-coupled Markov chain Monte Carlo (MCMCMC) method in MrBayes v. 3.2.6 on the CIPRES Web Portal^68^. Best-fit evolutionary models were estimated using Akaike Information Criterion (AIC^69^) as implemented in jModelTest2 2.1.6^70^. The TVM + I + G and the HKY + G models were selected respectively for the mtSSU and the nuITS datasets. For each of them, two parallel MCMCMC runs were performed, each using four independent chains and 20 million generations, sampling trees every 1000^th^ generation. Tracer v. 1.6^71^ was used to ensure that stationarity was reached by plotting the log-likelihood values of the sample points against generation time. Convergence between runs was also verified using the PSRF (Potential Scale Reduction Factor), where all values were equal or close to 1.000. Posterior probabilities (PP) were determined by calculating a majority-rule consensus tree generated from the 30002 post-burnin trees of the 40002 trees sampled by the two MCMCMC runs using the sumt option of MrBayes. In addition, a Maximum Likelihood (ML) analysis was performed using RAxML-HPC2 v.8.2.10^72^ with 1000 ML bootstrap iterations (ML-BS) and the GTRGAMMA model.

The RAxML tree did not contradict the Bayesian tree topology for the strongly supported branches. Therefore, only the RAxML tree is shown for the mtSSU dataset with the bootstrap support values and the posterior probabilities of the Bayesian analysis added near the internal branches (Fig. 1), whereas only the Bayesian tree is shown for the nuITS dataset with the posterior probabilities and the bootstrap support values of the RAxML analysis added near the internal branches (Fig. 2). ML-BS ≥ 70 and PP ≥ 0.95 were considered to be significant. Phylogenetic trees were visualized using FigTree v. 1.4.2^73^.

### Phylogenetic analyses of trebouxioid algae

In case of algal nuITS rDNA region, the newly obtained sequences were aligned together with *Trebouxia* sequences generated in the study of Voytsekhovich & Beck^36^ which are deposited in GenBank as PopSet 940816236 and additionally with best-hits from BLAST searches of newly sequenced photobionts, i.e. *Trebouxia* sp. TR9 (KU716051) and *Trebouxia* sp. P-400-IaSc (AM159210), using MAFFT on XSEDE (7.305) on the CIPRES Web Portal^68^. It was followed with a selection of ambiguous positions using Gblocks 0.91b^74,75^ using less stringent settings (i.e. allow smaller final blocks, gap positions within the final blocks and less strict flanking positions). The excluded regions were the terminal ends corresponding to positions 1–2632 and 3300–7640 of the sequence KU716051 (*Trebouxia* sp. TR9) and positions 1–689 of the sequence AM159210 (*Trebouxia* sp. P-400-IaSc). Moreover, the excluded regions were mainly due to insertions, i.e. in *Trebouxia gelatinosa* (e.g. positions 57–253 of the sequence KT819972), *T*. *arboricola* and *T*. *crenulata* (e.g. positions 158–189 of the sequence KT819921), *T*. *gigantea* and *T*. *incrustata* (e.g. positions 458–543 of the sequence KT819922), *T*. *arboricola*, *T*. *crenulata*, *T*. *decolorans*, *T*. *solaris* and *Trebouxia* sp. P-400-IaSc (e.g. positions 581–599 of the sequence KT819921).

Bayesian analyses were carried out for algal nuITS dataset using the Metropolis-coupled Markov chain Monte Carlo (MCMCMC) method in MrBayes v. 3.2.6 on the CIPRES Web Portal^68^. GTR + I + G best-fit evolutionary model was selected based on Akaike Information Criterion (AIC^69^) as implemented in MrModelTest2^76^. Two parallel MCMCMC runs were performed, each using four independent chains and 20 million generations, sampling trees every 1000th. Tracer v. 1.6^71^ was used to ensure that stationarity was reached by plotting the log-likelihood values of the sample points against generation time. Convergence between runs was also verified using the PSRF (Potential Scale Reduction Factor), where all values were equal or close to 1.000. Posterior probabilities (PP) were determined by calculating a majority-rule consensus tree generated from the 60002 post-burnin trees of the 80002 trees sampled by the two MCMCMC runs using the sumt option of MrBayes. In addition, a Maximum Likelihood (ML) analysis was performed using RAxML-HPC2 v.8.2.10^72^ with 1000 ML bootstrap iterations (ML-BS) and the GTRGAMMA model.

The RAxML tree did not contradict the Bayesian tree topology for the strongly supported branches, therefore, only the Bayesian tree is shown for algal nuITS rDNA dataset with the bootstrap support values and posterior probabilities of the Bayesian analysis added near the branches (Fig. 3). ML-BS ≥ 70 and PP ≥ 0.9 were considered to be significant and are shown near the branches.

### Phylogenetic analyses of trentepohlioid algae

For the trentepohlioid *rbcL* gene analysis, the newly generated sequences were aligned together with all sequences of *Trentepohliales* (200 records) available in GenBank using Seaview software^77,78^ employing muscle option and excluding ambiguous positions in Gblocks 0.91b^74,75^ using less stringent settings (i.e. allow smaller final blocks, gap positions within the final blocks and less strict flanking positions). The preliminary Bayesian analysis was carried out using the Metropolis-coupled Markov chain Monte Carlo (MCMCMC) method in MrBayes v. 3.2.6 on the CIPRES Web Portal^68^. Based on the tree obtained, we decided to reduce our dataset to 60 sequences using selected representatives (one or two sequences) of all main lineages. Moreover, sequences that were significantly shorter were also excluded from analyses. Terminal ends of the alignment corresponding to positions 1–321 and 990–1427 in the sequence KM464712 of *Trentepohlia annulata* were excluded from the alignment.

Bayesian analyses were carried out for trentepohlioid *rbcL* dataset using the Metropolis-coupled Markov chain Monte Carlo (MCMCMC) method in MrBayes v. 3.2.6 on the CIPRES Web Portal^68^. GTR + I + G best-fit evolutionary model was selected based on Akaike Information Criterion (AIC^69^) as implemented in MrModelTest2^76^. Two parallel MCMCMC runs were performed, each using four independent chains and 10 million generations, sampling trees every 1000th. Tracer v. 1.6^71^ was used to ensure that stationarity was reached by plotting the log-likelihood values of the sample points against generation time. Convergence between runs was also verified using the PSRF (Potential Scale Reduction Factor), where all values were equal or close to 1.000. Posterior probabilities (PP) were determined by calculating a majority-rule consensus tree generated from the 15002 post-burnin trees of the 20002 trees sampled by the two MCMCMC runs using the sumt option of MrBayes. In addition, a Maximum Likelihood (ML) analysis was performed using RAxML-HPC2 v.8.2.10^72^ with 1000 ML bootstrap iterations (ML-BS) and the GTRGAMMAI model.

The RAxML tree did not contradict the Bayesian tree topology for the strongly supported branches, therefore, only the Bayesian tree is shown for trentepohlioid *rbcL* dataset with the bootstrap support values and posterior probabilities of the Bayesian analysis added near the branches (Fig. 4). ML-BS ≥ 70 and PP ≥ 0.9 were considered to be significant and are shown near the branches.

Phylogenetic trees were visualized using FigTree v. 1.4.2^73^.

The DNA sequences generated during the current study are available in the GenBank repository (https://www.ncbi.nlm.nih.gov; see Table 1) and from the corresponding author on reasonable request.
